# An analysis of B-cell epitope discontinuity

**DOI:** 10.1016/j.molimm.2012.03.030

**Published:** 2012-07

**Authors:** Ganesh N. Sivalingam, Adrian J. Shepherd

**Affiliations:** a Research Department of Structural and Molecular Biology, Institute of Structural and Molecular Biology, University College London, Gower Street, London WC1E 6BT, UK; b Department of Biological Sciences, Institute of Structural and Molecular Biology, Birkbeck, University of London, Malet Street, London WC1E 7HX, UK

**Keywords:** ASA, accessible surface area, PDB, Protein Data Bank, Antigen, Antibody, Epitope, Structural epitope, Functional epitope

## Abstract

Although it is widely acknowledged that most B-cell epitopes are discontinuous, the degree of discontinuity is poorly understood. For example, given that an antigen having a single epitope that has been chopped into peptides of a specific length, what is the likelihood that one of the peptides will span all the residues belonging to that epitope? Or, alternatively, what is the largest proportion of the epitope's residues that any peptide is likely to contain? These and similar questions are of direct relevance both to computational methods that aim to predict the location of epitopes from sequence (linear B-cell epitope prediction methods) and window-based experimental methods that aim to locate epitopes by assessing the strength of antibody binding to synthetic peptides on a chip.

In this paper we present an analysis of the degree of B-cell epitope discontinuity, both in terms of the structural epitopes defined by a set of antigen–antibody complexes in the Protein Data Bank, and with respect to the distribution of key residues that form functional epitopes. We show that, taking a strict definition of discontinuity, all the epitopes in our data set are discontinuous. More significantly, we provide explicit guidance about the choice of peptide length when using window-based B-cell epitope prediction and mapping techniques based on a detailed analysis of the likely effectiveness of different lengths.

## Highlights

► All the structural B-cell epitopes we examined are discontinuous. ► Only 18% of structural epitopes are spanned by a peptide fragment of 40 residues. ► Centralized and random distributions were considered for key functional residues. ► Fragments with only 7 residues will successfully span most key functional residues.

## 1 Introduction

### 1.1 B-cell epitope identification

It is widely recognized that knowledge about B-cell epitopes is important for the identification or design of therapeutic antibodies, and for gaining insights into vaccine effectiveness (Irving et al., 2001). Various methods may be used to determine – with varying degrees of accuracy – the location of B-cell epitopes, ranging from purely computational methods to X-ray crystallography. (For a useful survey of methods, see Ladner (2007).)

Whereas approaches such as X-ray crystallography and site-directed mutagenesis are capable of determining the location of B-cell epitopes with a high degree of accuracy, the efficacy of the methods we focus on here – computational methods for predicting the location of linear B-cell epitopes and short peptide mapping techniques – is somewhat uncertain. Nevertheless, these methods have an enduring appeal, as they are comparatively cheap and can be used as the basis for high-throughput screening, properties that more accurate methods do not possess.

A range of *computational methods* have been developed for predicting which of an antigen's residues are likely to form part of an epitope (El-Manzalawy and Honavar, 2010). In the absence of useful structural information about the antigen, predictions must be made using the primary amino-acid sequence alone. Typically a fixed-length profile is generated from a set of known examples and applied to a given antigen using a sliding window.

Such methods are primarily suited to find linear B-cell epitopes, i.e. epitopes that consist of a single more-or-less continuous segment from the primary sequence. But this begs the questions: How strict does the definition of “continuous” have to be? And what proportion of epitopes meet these requirements in practice?

*Short peptide mapping* involves the synthesis of relatively short overlapping peptides from the antigen of interest and measuring the extent to which they bind to a given antibody. The peptide may be in linear conformation, or constrained in some way to mimic, to some degree, the 3-dimensional conformation of that peptide in its natural (*in vivo*) structural context (Timmerman et al., 2009). Given an antigen of interest, it is up to the researcher to decide how to split it into individual peptides. In practice, experimentalists typically choose a fixed window size (peptide length) and shift that window by a fixed amount along the full length of the antigen sequence (maintaining a consistent degree of overlap). However, the window size and degree of shift can vary significantly between different experiments. For example, Geysen et al. (1984) chose a window of size six and shifted the window by a single position (hence an overlap of five), whereas Behan et al. (1998) used a window of size 17 shifted by five residues (hence an overlap of 12). Peptides of up to 32 residues were used by Timmerman et al. (2007), but such large window sizes are exceptional.

Note that in this paper we deliberately exclude from consideration variations on these peptide-mapping approaches that model discontinuous epitopes by combining non-adjacent segments from a protein sequence. To be effective, such approaches generally require significant prior knowledge about the location of epitope residues – see, for example, the analysis of CD20 antibodies in Niederfellner et al. (2011).

Before considering whether these epitope prediction and small peptide mapping approaches have inherent limitations, it is essential to consider what is known about the properties of B-cell epitopes.

### 1.2 Properties of B-cell epitopes

There are various ways of defining what an “epitope” is (see Ladner, 2007), but probably the most widely used definition is that of a *structural epitope*. A structural epitope consists of the set of the antigen's amino-acid residues that are in direct contact with residues belonging to an antibody (the paratope).

Several fundamental properties of structural epitopes have been quantified in an analysis of 53 antigen–antibody complexes from the Protein Data Bank (PDB) (Berman et al., 2000) undertaken by Rubinstein et al. (2008). For example, the study concluded that approximately 75% of epitopes consist of 15–25 residues with a surface area of 600–1000 Å^2^. They also partially quantified the degree to which B-cell epitopes are discontinuous. No epitopes in their data set were found to be strictly linear, i.e. composed of a single, continuous segment of the antigen's amino-acid sequence having all residues in direct physical contact with one or more antibody residues. Using a less strict criterion that allowed up to three non-contact residues to occur within a segment, the authors found that most epitopes consist between one and five segments, each containing one to six residues.

Whereas the definition of a structural epitope is widely used and easy to grasp, it is not necessarily the most relevant for the purpose of epitope mapping. On the one hand, some non-contact residues have been shown to induce conformational changes that affect antigen–antibody binding (Parry et al., 1990); on the other hand, it is widely recognized that, in general, only a subset of contact residues within an epitope make a significant contribution to the global binding energy (Novotny, 1991). These energetically important residues – which typically number between three and five, and which can be determined experimentally using site-directed mutagenesis (Benjamin and Perdue, 1996) – are commonly known as hot spot residues and collectively form a so-called *functional epitope*.

The properties of protein–protein interfaces in general have been widely characterized in the literature; a small number of hot-spot residues account for most of the binding energy (Bogan and Thorn, 1998) and are grouped in one or a few “hot regions” towards the centre of the interface (Keskin et al., 2004). However, whereas some authors assume there is nothing special about B-cell epitopes – indeed, the term epitope is sometimes used loosely to refer to any protein interface (see, for example, Ma et al., 2001) – this assumption may not be justified, as there are important differences between the binding characteristics of antigen–antibody complexes and those of other classes of complex. For example, Jackson found that serine protease–inhibitor complexes involve backbone interactions, whereas side-chain interactions dominate in antigen–antibody complexes (Jackson, 1999).

All things considered, we should expect antigen–antibody interfaces to be a special case. Whereas other protein–protein interfaces will typically have evolved cooperatively (with both partners making a complementary contribution), in antigen–antibody interfaces the antigen is either passive or actively evolves to resist the formation of the complex. Whereas other protein–protein interfaces are likely to be mature (established over significant periods of time), antigen–antibody interfaces are often transients (witness, for example, the short-lived effectiveness of most antibodies against the evolving influenza A virus (Wilson and Cox, 1990)).

### 1.3 The limitations of window-based methods

Some of the current limitations of window-based computational prediction methods and peptide mapping techniques have already been discussed elsewhere. Blythe and Flower (2005) demonstrated that simple sequence profiles based on a single propensity scale are little better than random at predicting the location of linear epitopes. For short peptide mapping techniques, the likely conformational differences between a given peptide and the corresponding region from the intact protein have been widely acknowledged (Van Regenmortel, 2006; Chen et al., 2009).

But arguably there remains an even more fundamental question: for methods that utilize relatively small windows onto the primary amino-acid sequence, how likely is it that a segment will be found that spans a significant number of epitope residues? This is one of the questions we address in this paper. More generally, we seek to extend the analysis already carried out by Rubinstein et al. (2008) into the properties of structural epitopes by quantifying the degree to which B-cell epitopes – both structural and functional – are discontinuous.

## 2 Methods

### 2.1 Protein data set

A dataset of X-ray crystallographic antigen–antibody structures was constructed based on an initial list derived from the Summary of Antibody Crystal Structures (SACS) database (Allcorn and Martin, 2002). Various criteria were imposed to filter out inappropriate structures, notably those with missing data or of low quality. Hence structures were removed that did not have:

• A resolution of ≤3 Å.
• An antibody component comprising at least part of both heavy and light chains.
• An antigen component containing at least 30 amino-acid residues.
• And a complete structural epitope (i.e. no missing information relating to the antigen's epitopic residues).

We also excluded structures where the epitope includes residues from multiple chains of the antigen. Such epitopes are relatively uncommon (accounting for only 2.6% of those selected according to the preceding criteria), although they are potentially important in specific contexts; for example, epitopes that span the HA1 and HA2 domains in influenza A haemagglutinin are at the centre of research seeking to identify the targets for vaccines with long-term effectiveness (Ekiert et al., 2009; Corti et al., 2011). However, such epitopes are hard or impossible targets for the window-based methods we are concerned with here, which consider only continuous peptides, and including them would have over-complicated our analyses.

Given the need to automate the selection of appropriate structures for our data set, various heuristics were implemented (based on Li and Wang (2009)) to exclude PDB entries for which we were unable to reliably extract information in a relatively straightforward manner. For example, we excluded any structure for which we were unable to identify the antibody chains using simple keywords (such as “heavy”, “H”, “light”, “L” and “IG”).

Having applied these criteria, we ended up with a data set consisting of 150 antigen–antibody complexes.

### 2.2 Structural epitope data set

Within our set of complexes, an individual residue forming part of the antigen was deemed to be part of the structural epitope provided its accessible surface area (ASA) increases when the antibody is removed from the complex. ASA was calculated by applying the Shrake–Rupley algorithm (Shrake and Rupley, 1973).

This approach is broadly similar to that adopted by a number of authors (Ponomarenko and Bourne, 2007; Rubinstein et al., 2008), but different approaches are possible. For example, a distance cut-off of 6 Å is used to define epitopes for the Epitome database (Schlessinger et al., 2006), whereas Sobolev et al. (1999) exclude from consideration putative interactions involving atom pairings that are electrostatically unfavourable (e.g. between an aliphatic carbon and a carbonyl oxygen). In a preliminary comparison of the effect of using different criteria for defining epitope residues on a set of six structures (results not shown), the criteria we have adopted differed by only 1.5% from those used in the study by Rubinstein et al. (2008), which represents the closest comparable study to our own.

### 2.3 Functional epitope data sets

Although there are some examples of functional epitopes for which the precise residues are known, the number is comparatively small. Here we analyse a small set of known functional epitopes, derived from the data set in Duquesnoy (2006), but our main analysis focuses on two sets of simulated functional epitopes derived from our structural data set. These two sets represent the two extremes of how the residues of a functional epitope might be distributed with respect to the corresponding structural epitope. In the first set, all functional residues are assumed to be clustered in a single patch located centrally within the corresponding structural epitope; in the second set, the functional residues are assumed to be randomly distributed across the structural epitope as a whole.

With both these data sets, functional epitopes having three and five residues were assessed. For the random set, results for a given functional epitope having a set number of residues were calculated as follows. All unique combinations of that number of residues within the set of residues forming the corresponding structural epitope were calculated. In the case where there were fewer than 100 such combinations, all were selected; when there were more than 100 combinations, 100 were selected from the full set at random. Analysis was carried out on the combined set of randomized variants from all structures.

## 3 Results

### 3.1 Structural epitope segmentation

As in the earlier data set of Rubinstein et al. (2008), none of the epitopes in our structural data set are strictly linear, i.e. with no gaps permitted between the epitopic residues in direct contact with the antibody. However, this is an excessively stringent requirement for many purposes. For example, for small peptide mapping methods to be successful, it is probably necessary that a significant percentage of key epitopic residues occur within a peptide that maps onto that epitope, but that does not mean that a continuous stretch of residues within the peptide need to map to contact residues.

**Fig. 1:**
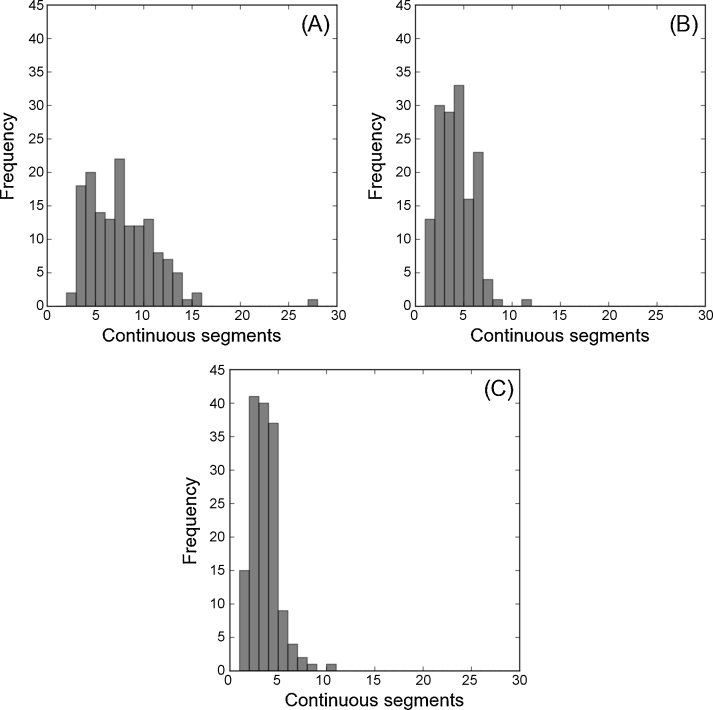
The segmentation of structural epitopes using different definitions of a continuous segment. (A) No gaps are tolerated within a single segment. (B) Gaps of up to three sequential non-epitope residues are tolerated within a single segment. (C) Gaps of up to five sequential non-epitope residues are tolerated within a single segment.

Here we adopt a similar approach to that used by Rubinstein et al. (2008) by progressively relaxing the definition of a continuous segment (Fig. 1). In Fig. 1(A), a strict definition of continuous segment is applied, i.e. a gap of a single non-epitope residue is sufficient for the sequence to be split into two segments. In Fig. 1(B), one or more gaps of up to three consecutive non-epitope residues are tolerated, but not a gap of four or more non-epitope residues. And in Fig. 1(C) one or more gaps of up to five consecutive non-epitope residues are tolerated.

One striking feature of these results is that, even when we allow generous gaps of up to five non-epitope residues to occur within a single segment (Fig. 1(C)), a substantial majority (85%) of epitopes have multiple segments. With a less generous gap of up to three non-epitope residues within a segment, 88% of structures have multiple segments.

### 3.2 Preliminary analysis of functional epitopes

In Table 1 we summarize the characteristics of six experimentally determined functional epitopes presented in Duquesnoy (2006) that are associated with known structural epitopes.^1^ Duquesnoy points out that the functional epitopes associated with three structures (PDB ID: 1VFB, 3HFL, 1JRH) consist of a single patch located centrally within the corresponding structural epitope, whereas the functional epitopes associated with the remaining three structures (PDB ID: 1HFM, 1FBI, 1WEJ) comprise two distinct patches.

In terms of the questions we address in this paper, what we are interested in is the number of residues that occur within a functional epitope and how they are distributed with respect to the underlying primary sequence. In this respect, the results in Table 1 are instructive, in spite of the small sample size. On the one hand, the number of residues making up a given functional epitope does not appear to be highly correlated with the length of the shortest peptide that spans all the residues belonging to that functional epitope; hence the functional epitope with the shortest spanning peptide has the largest number of functional residues (PDB ID: 1JRH). On the other hand, there does appear to be a significant relationship between the length of the shortest spanning peptide and the way functional residues are distributed in terms of the formation of contiguous patches in three-dimensional space; hence the average length of the minimum spanning peptide for functional epitopes consisting of a single patch (12 residues) is much shorter than that for functional epitopes in which the residues are clustered in more than one patch (66.7 residues).

In the absence of a public database of functional epitopes, we have chosen to derive two sets of simulated functional epitopes – each containing simulated epitopes with three and five key functional residues – that map onto known structural epitopes in contrasting ways: either clustered in a single centrally located patch, or distributed randomly (see Section 2.3).

### 3.3 Minimal spanning peptide

**Fig. 2:**
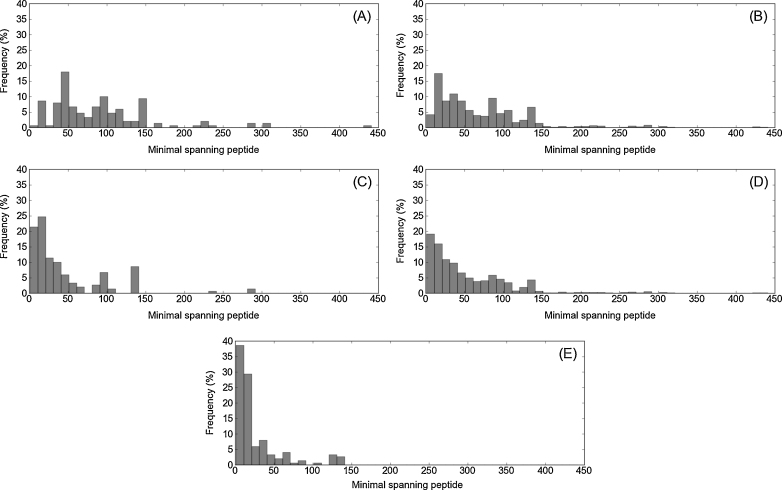
Histograms (with bins of size 10) of the lengths of the minimal spanning peptides for the epitopes in our dataset. (A) Structural epitopes. (B) Functional epitopes comprising 5 randomly located residues. (C) Functional epitopes comprising 5 centrally located residues. (D) Functional epitopes comprising 3 randomly located residues. (E) Functional epitopes comprising 3 centrally located residues.

For any single-chain structural or functional epitope, there is a corresponding minimal peptide (consisting of a continuous sub-sequence of the full amino-acid sequence for that chain) that spans all the contact residues belonging to that epitope. In Fig. 2, we have plotted the lengths of the minimal spanning peptides in a histogram.

Fig. 2(A) is sobering for those hoping to capture all the residues in a structural epitope within a single synthetic (or computationally modelled) peptide of even moderate length, as the number of epitopes for which all epitopic residues are captured with a peptide of length 20 is less than 10%. When the peptide length is raised to 40 – which is significantly longer than all the small peptide mapping experiments we are aware of – the percentage rises to only 18%.

The same analyses performed for our artificial sets of functional epitopes give results that are more promising for window-based methods. In the worst-case scenario (Fig. 2(B)), with functional epitopes consisting of five residues that are randomly distributed with respect to the corresponding structural epitope, 21% are captured by peptides of length 20 and 40% by peptides of length 40. A more realistic opportunity for window-based methods to capture all the desired information arises when the residues of functional epitopes form a single central patch. With five such residues (Fig. 2(C)), peptides of length 20 capture 47% of the epitopes, and peptides of length 40 capture 69%; with only three such residues (Fig. 2(E)) – the best-case scenario for window-based methods – peptides of length 20 capture 67% of the epitopes, and peptides of length 40 capture 80%.

Even in the best-case scenario, these results are rather discouraging for advocates of window-based epitope detection methods. Commonly used peptide lengths of 7–15 residues will fail to capture all the key functional residues in about 50% of cases – even when the functional epitopes comprise only three residues that are adjacent to each other in three-dimensional space. But there remains one redeeming possibility: that, in practice, it is necessary to capture some – but not all – of the key functional residues.

### 3.4 Epitope coverage given windows of different sizes

**Fig. 3:**
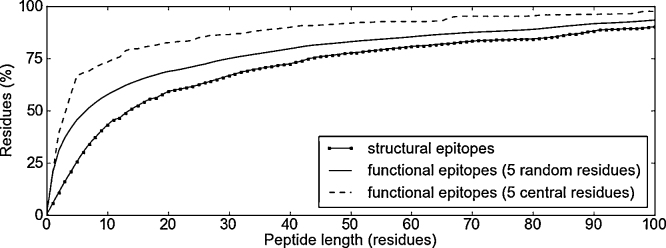
Graphs showing the average percentage of epitope residues (*y*-axis) capture by peptides of different lengths (*x*-axis). The score is averaged across the best spanning peptides for all epitopes in the given data set, where the best spanning peptide (of a given length) for a single epitope is the one that spans the greatest number of that epitope's residues (compared to other peptides of the same length).

For window-based methods, whether computational or experimental, a key question is: how much of an epitope am I likely to encompass, on average, for a given window size/peptide length? The answer to this question is given in Fig. 3 for structural epitopes, functional epitopes with five randomly distributed residues, and functional epitopes with five residues that form a single, central patch.

These results show that windows of the size typically used in small peptide experiments (7–15 residues) capture roughly 35–55% of structural epitope residues, 50–65% of residues in functional epitopes when the residues are randomly distributed, and 70–80% of residues when the functional residues form a single patch.

## 4 Discussion

It is easy to understand the enduring appeal of window-based techniques – both computational and experimental – for identifying the location of B-cell epitopes. Experimental techniques of high accuracy, such as X-ray crystallography and site-directed mutagenesis, are much more time consuming and expensive. But doubts remain about the true efficacy of window-based methods, and there is a persistent risk of reporting bias; in other words, a risk that the apparent successes of such methods will be reported in the literature, whereas their failures will not.

In this paper we have sought to shed light upon the intrinsic limitations of a range of computational and experimental window-based techniques given their reliance on linear peptides of modest length. We have concluded our analysis on a relatively positive note (see Section 3.4). In the most favourable scenario where, for a method to successfully locate a B-cell epitope, it needs to only detect three or four residues from a single patch comprising a small set of key binding residues, our results give significant grounds for optimism; in most cases, there exists a peptide of modest length (of 15 residues or less) that will span at least that number of residues.

However, not all epitopes have a single-patch of key functional residues; our results suggest that epitopes having functional residues that are more widely distributed will often be missed by methods that rely on short linear peptides. Moreover, it may transpire that a past reliance on window-based approaches to detect epitopes is reflected in a degree of bias towards epitopes that are detectable by such approaches within the PDB itself.

We should also end with a further note of caution. In order for an epitope to be successfully detected using any of the window-based techniques considered in this paper, it is probably necessary for there to exist, within the full protein sequence, a comparatively short peptide spanning most of that epitope's key functional residues. However, the existence of such a peptide is not necessarily sufficient. For short peptide mapping techniques, the conformation of such a peptide in three-dimensional space is likely to play a vital role in their success or failure, but this is a topic that lies outside the scope of this paper.

## Figures and Tables

**Table 1:** Comparison of structural and functional characteristics of six epitopes.

PDB code	Structural epitope^a^		Functional epitope^b^		
	Number of residues	Span	Number of residues	Span	Number of patches
1VFB	23	117	4	7	1
3HFL	21	44	2	24	1
1HFM	24	95	4	78	2
1FBI	26	89	3	77	2
1WEJ	18	102	4	45	2
1JRH	17	53	5	5	1

a All numbers calculated by the authors.

b Span calculated by the authors, other numbers taken from Duquesnoy (2006).

## Glossary

Discontinuous epitope: An epitope that is not a linear epitope, i.e. an epitope that consists of multiple, distinct segments from the primary amino-acid sequence.
Functional epitope: The subset of residues belonging to an epitope that accounts for most of the binding energy in the antigen–antibody complex. Typically there are between three and five such residues.
Linear epitope: Strictly speaking, an epitope consisting of a single continuous segment from the primary amino-acid sequence. However, less stringent definitions that allow the continuity of the segment to be broken by a small number of non-epitope residues are widely used.
Short peptide mapping technique: A technique for locating the epitopes of a given antigen that involves synthesizing a series of short overlapping peptides from that antigen's primary sequence and measuring which ones bind to an antibody.
Structural epitope: The set of amino-acid residues belonging to an antigen that is in direct contact with a corresponding set of residues belonging to an antibody.

## Footnotes

1 A seventh structure from Duquesnoy (2006) (PDB ID: 1DQJ) has been omitted on the grounds that its functional epitope shares two out of three epitopic residues with one of our chosen structures (PDB ID: 1FBI).
